# Protective Effect of Resveratrol Improves Systemic Inflammation Responses in LPS-Injected Lambs

**DOI:** 10.3390/ani9110872

**Published:** 2019-10-28

**Authors:** Yanping Liang, Jianwei Zhou, Kaixi Ji, Hu Liu, Allan Degen, Manjun Zhai, Dan Jiao, Junqiang Guo, Zongsheng Zhao, Guo Yang

**Affiliations:** 1 College of Animal Science and Technology, Shihezi University, Shihezi 832003, China; liangyp1045@yahoo.com (Y.L.); zhaimanjun@yeah.net (M.Z.); 2 Northwest Institute of Eco-Environment and Resources, Chinese Academy of Sciences, Lanzhou 730000, China; zhoujw@lzb.ac.cn (J.Z.); ji_kaixi@163.com (K.J.); tigerliu18@163.com (H.L.); jiaodan@lzb.ac.cn (D.J.); guojunqlzb@163.com (J.G.); 3 Desert Animal Adaptations and Husbandry, Wyler Department of Dryland Agriculture, Blaustein Institutes for Desert Research, Ben-Gurion University of the Negev, Beer Sheva 8410500 Israel; degen@bgu.ac.il

**Keywords:** resveratrol, lipopolysaccharide (LPS), inflammation responses, cytokines, lamb

## Abstract

**Simple Summary:**

China’s livestock industry has been transforming from traditional extensive systems to highly intensive systems. Highly intensive livestock production often causes immune stress to animals, which makes them more susceptible to infections. The aim of this study was to examine whether resveratrol alleviates inflammation in lambs. Results showed that resveratrol attenuated the LPS-evoked inflammatory responses in lambs by suppressing expression levels of inflammatory cytokines and blocking *NF-κB* and MAPK signaling pathways. Based on these studies, resveratrol has the potential to be a promising therapeutic reagent for multiple inflammatory illnesses caused by immune stress.

**Abstract:**

Highly intensive livestock production often causes immune stress to animals, which makes them more susceptible to infections. The aim of this study was to examine whether resveratrol (Res) alleviates inflammation in lambs. In Experiment 1, 16 male lambs were injected with lipopolysaccharides (LPS) at an initial dose of 0.25, 1.25, and 2.5 μg/kg body weight (BW) for 9 days. Average daily gain and blood parameters were measured and clinical symptoms were recorded. In Experiment 2, 20 male lambs were injected intravenously with LPS (0 mg/kg) + Res (0 mg), LPS (2.5 μg /kg) + Res (0 mg, 82.5 mg, 165 mg, 330 mg), 4 h after LPS injection. Jugular blood was collected from each lamb to determine white blood cell (WBC) counts and the expression of inflammatory genes. In Experiment 1, all LPS-treated lambs showed clinical signs of sickness including rhinorrhea, lethargy, and shivering, and systemic inflammatory responses of increased inflammatory genes levels and cortisol concentration. The lambs had increased respiratory and heart rates and rectal temperature and decreased average daily gain and feed intake. In Experiment 2, resveratrol significantly reduced WBCs and the expression levels of several genes associated with inflammation response (*TLR4*, *NF-κB*, *c-jun*) and inhibited the signaling cascades of *NF-κB* and MAPKs by down-regulating the expression levels of inflammatory cytokines (*IL-1β*, *IL-4*, *IL-6*, *TNF-α*, *IFN-γ*) induced by LPS. Resveratrol attenuated the LPS-evoked inflammatory responses in lambs by suppressing expression levels of inflammatory cytokines, and blocking *NF-κB* and MAPK signaling pathways.

## 1. Introduction

China’s livestock industry has been transforming from traditional extensive systems to highly intensive systems. Livestock are vulnerable to immune stress under intensive conditions, which can lead to infections and outbreaks of animal epidemics [1]. 

Intravenous injection of bacterial lipopolysaccharides (LPS) simulates infection in livestock and can be used for the study of immune inflammatory responses without the risk of using a live pathogen [2,3,4]. LPS is a major component of the outer membrane of gram-negative bacteria such as *Escherichia coli* and a chief member of the pathogen-associated molecular patterns [5]. It binds to the CD14/TLR4/MD2 receptor complex initiating the activation of intracellular signaling pathways, including mitogen-activated protein kinases (MAPK) and nuclear factor *κB* (*NF-κB*). It also stimulates the expression of inflammatory mediators, including interleukin-1β (*IL-1β*), tumor necrosis factor alpha (*TNF-α*), interleukin-6 (*IL-6*), and interferon gamma (*IFN-γ*), causing pathological reactions such as inflammation, fever, and shock to the body [3,6]. The pathogenesis of many diseases, including inflammatory bowel disease, sepsis and cancer, involves inflammation [7]. Consequently, inhibition of the expression of inflammatory factors or inflammatory genes is an important target for preventing or treating various diseases.

Resveratrol (trans-3, 4’, 5-trihydroxystilbene), a natural plant phytoalexin, is widely distributed in many plants. It occurs in high concentrations in grape skins, peanuts and wines and possesses anti-inflammatory, anti-cancerogen and antioxidant properties [8,9,10,11]. Studies involving resveratrol treatment in animals with inflammatory diseases have demonstrated downregulation of inflammation-induced biomarkers, including pro-inflammatory mediators (*IL-1β*, *IL-6*, *TNF-α*, *MCP-1*, *IFN-γ*, and *NF-κB*) [12,13,14,15] and upregulation of inflammation-reduced biomarkers, including anti-oxidant protein (SOD) and total WBC counts [16,17]. Calabrese et al. [18] classified resveratrol as a hormetic modulator in diseases, which included inflammation and infections.

Anti-inflammatory effects of resveratrol have been largely confirmed in rodents, but there have been few studies on livestock. To fill this gap, the aim of this study was to measure responses to the administration of resveratrol in LPS-challenged lambs. Lambs were chosen because intensive sheep production is an important and expanding enterprise in China and lambs are very susceptible to diseases. These data are necessary prior to consideration of resveratrol as an immunomodulatory intervention agent in clinical practice and as a potential feed additive in livestock production systems. 

## 2. Materials and Methods

All experimental procedures were in accordance with the animal welfare legislation and approved by the Academic Committee of Northwestern Institute of Eco-Environment Resources, Chinese Academy of Sciences (protocol number: CAS201810082). The animals were supplied by the Gaolan Ecological and Agricultural Integrated Experimental Station in Gansu, China.

### 2.1. Experiment 1

Sixteen healthy male Hu lambs (4 to 5 months of age and 22.2 ± 0.52 kg BW) were penned individually with free access to food and water. Composition and energy yield of the diet are presented in Table 1. Air temperature was maintained at 13 ± 1.4 °C and relative humidity at 81% ± 9.5%. After 5 days of adaptation to the conditions, each lamb received a jugular injection of LPS isolated from *Escherichia coli O111:B4* (Sigma, St. Louis, MO, USA) at 08:00 on days 1, 3, 5, 7 and 9 to induce a chronic inflammatory response [2,4]. Initial doses of 0 (control group, n = 4), 0.25 (LPSL group, n = 4), 1.25 (LPSM group, n = 4) and 2.5 (LPSH group, n = 4) µg LPS/kg BW were injected, which were increased by 20% at each subsequent injection. Lambs are sensitive to LPS [19], and, consequently, the increase was only 20% to avoid the risk of sensitization and mortalities. 

The lambs were weighed at the beginning and at the end of the experiment (days 0 and 10). Dry matter intakes were recorded daily by weighing the feed offered and the feed remains. Rectal temperature of each lamb was measured 3 h after LPS injection.

Ten mL jugular vein blood samples were collected in tubes with EDTA before morning feeding on day 0 and 3 h after the LPS challenge on days 1, 3, 5, 7, and 9. White blood cells were counted in 2 mL of blood from each lamb on days 0, 1, 3, 5, 7, and 9 (Prang XFA6000 Automatic Blood Cell Analyzer, Nanjing, China). Five mL blood were centrifuged at 3500 rmp for 15 min at 4 °C, and plasma was stored at −80 °C until analysis. Another 5 mL of blood were used for RNA extraction.

### 2.2. Experiment 2

Twenty healthy male Hu lambs (5 months of age and 25.2 ± 0.54 kg BW) were penned individually and were offered free access to food and water. Air temperature was maintained at 5 ± 1.0 °C and relative humidity at 84 ± 4.4%. They were divided randomly into five groups (n = 4 per group) and after 5 days of adaptation to the conditions, each lamb received a jugular injection of resveratrol (Res; purity ≥ 98%, Shaanxi Xinzhikang Biological Technology Co., Ltd. Xi’an, China) diluted in 50% medical alcohol (Control: 0 mg, LPS: LPS + Res 0 mg; ResL: LPS + Res 82.5 mg; ResM: LPS + Res 165 mg; or ResG: LPS + Res 330 mg) daily at 08:00. LPS was isolated from *Escherichia coli O111:B4* (Sigma, St. Louis, MO, USA), diluted in saline and delivered via jugular injection at 08:30 on days 15 and 17. An initial dose of 2.5 µg LPS/kg BW was increased by 20% at each subsequent injection, whereas the lambs in the control group received an equal volume of saline and medical alcohol. Resveratrol is difficult to dissolve in normal saline but is soluble in medical alcohol and 50% medical alcohol has been used in studies [20]. For the purpose of this study, it was important to deliver very accurate amounts of resveratrol into the lambs; intrajugular injection was the most accurate method and, therefore, was employed.

Jugular vein blood samples (12 mL) were collected in vacuum tubes with EDTA at 4 h after resveratrol injection from each lamb on day 17 and white blood cells were counted in 2 mL whole blood (Prang XFA6000 Automatic Blood Cell Analyzer, Nanjing, China). Five mL of blood were centrifuged at 3500 rmp for 15 min at 4 °C, and the plasma was stored at −80 °C until analysis and another 5 mL of blood were used for RNA extraction. 

### 2.3. Enzyme Linked Immunosorbent Assays

Plasma cortisol concentration was measured using an enzyme-linked immunosorbent assay (ELISA) kit (Shanghai Elisa Biotech Co., Ltd., Shanghai, China) according to the manufacturer’s protocol, with some modifications. Briefly, 50 μL of standard/sample and 100 μL of horseradish peroxidase (HRP)-conjugate reagent were added to the antibody-coated 96-well plates, covered with an adhesive strip and then incubated for 60 min at 37 °C. The plates were then washed manually 4 times and chromogen solution A (50 μL) and chromogen solution B (50 μL) were added to each well, mixed gently and incubated for 15 min at 37 °C. Stop solution (50 μL) was then added and, within 15 min, the optical density was read at 450 nm using a microtiter plate reader.

### 2.4. Quantitative Real-Time PCR

The relative gene expression was quantified by quantitative real-time PCR (qPCR) using a qPCR kit (TB Green™ Premix Ex Taq™ II, TaKaRa, Tokyo, Japan), done on a Mx3000P Real-Time PCR System (Stratagene, La Jolla, CA, USA).

Total RNA was extracted from blood samples using TRIzol^®^ reagent (Invitrogen; ThermoFisher Scientific, Inc., Waltham, MA, USA) according to the manufacturer’s protocol. Reverse transcription was done on RNA with the FastKing RT Kit (with gDNase; TIABGEN Biotech Co. Ltd, Beijing, China). The mRNA expression levels of *IL-1β*, *IL-4*, *IL-6*, *IFN-γ*, *TNF-α*, *C3*, *TLR4*, *NF-κB*, and *c-jun* in the blood samples were detected by quantitative real-time PCR using a TB Green™ Premix Ex Taq™ II Kit (Tli RNaseH Plus) (TaKaRa, Tokyo, Japan) as specified by the manufacturer. The reference gene for normalization was β-actin. Primers (Table 2) were synthesized by AuGCT DNA-Syn Biotechnology Co., Ltd. (Beijing, China). The reaction was done in a 20 μL total reaction volume, which included 10 μL of the 2× TB Green™ Premix Ex Taq™ II, 0.4 μL of the ROX reference dye (50×), 0.8 μL each of the forward and reverse primers (10 μM), 2 μL of the cDNA template, and 6 μL of sterilized water. Relative fold changes in expression of candidate genes were obtained using the 2^−ΔΔCt^ method. The Ct values were used to calculate ΔCt values for genes of interest [Ct (test)−Ct(reference)]. All measurements were done in triplicate.

### 2.5. Statistical Analysis

Data are presented as means ± SD. One-way ANOVA was used to test for differences among groups using SPSS software version 17.0 (SPSS, Inc., Chicago, IL, USA). The Levene test was used to test for homogeneity of variances and the null hypothesis was that all variances were equal. A resulting *p*-value under 0.05 meant that variances were not equal. Dunnett’s test correction was used when the equality of variances assumption held, and Tamhane’s T2 post hoc test was used otherwise. Difference between means was accepted to be statistically significant at *p* < 0.05 and as a trend at 0.05 < *p* < 0.10. 

## 3. Results

### 3.1. Average Daily Gain, Feed Intake, and Clinical Symptoms after LPS-Challenge

**Figure 1:**
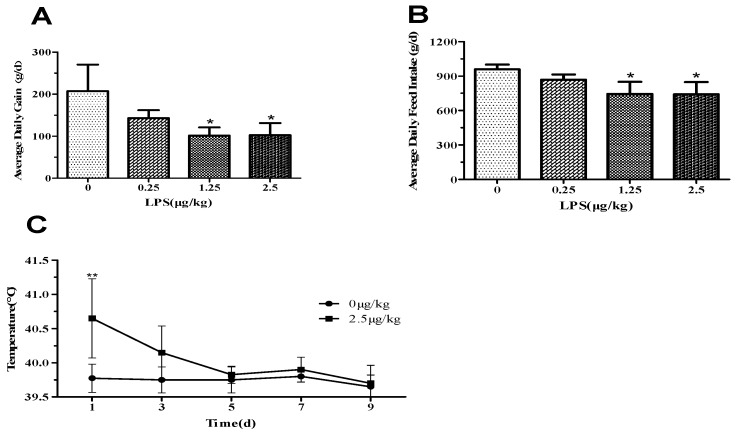
Effect of 2.5 μg /kg body weight lipopolysaccharides (LPS) on (**A**) average daily gain, (**B**) average daily feed intake and (**C**) rectal temperature in lambs. Values are expressed as means ± SD. * *p* < 0.05, ** *p* < 0.01, and *** *p* < 0.001, compared with the control group.

**Figure 2:**
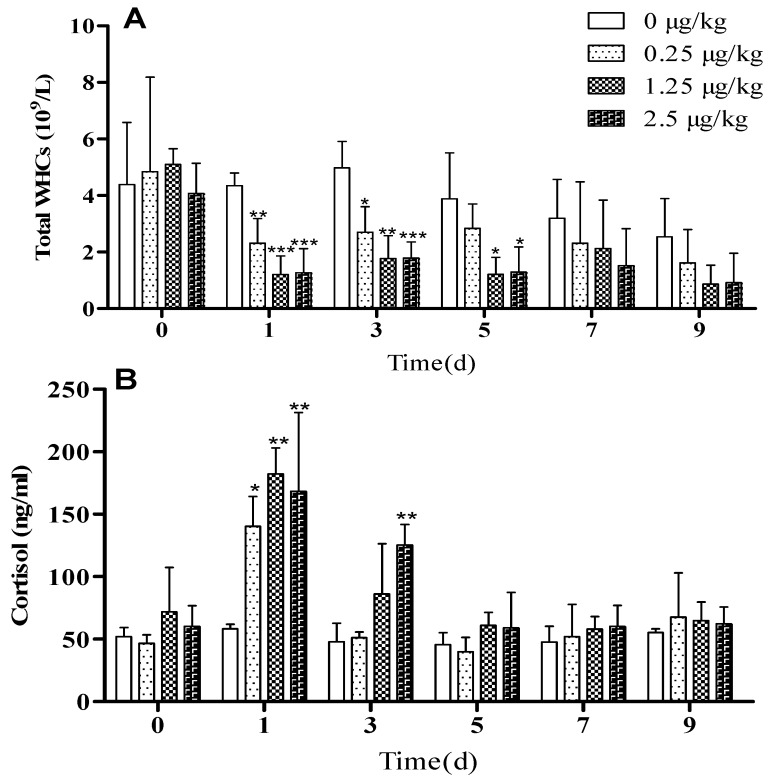
Effect of 0.25, 1.25, and 2.5 μg/kg LPS on (**A**) white blood cells (WBCs) and (**B**) plasma cortisol concentration in lambs. Values are expressed as means ± SD. * *p* < 0.05, ** *p* < 0.01, and *** *p* < 0.001, compared with the control group.

Within the first 3 hours after LPS administration, six lambs in the LPSM and LPSH groups had diarrhoea and, 60 minutes after administration on days 1 and 3, all lambs increased their respiratory rate, and seven lambs had rhinorrhea, became lethargic, and shivered. Heart rate in the LPSH group was significantly higher than in the control group (Supplementary Materials Table S1). Subsequent LPS injections resulted in lethargy, shivering, and hyperventilation in the lambs, but no diarrhoea was observed. The LPSM (*p* < 0.05) and LPSH (*p* < 0.05) groups had lower average daily gain (ADG; Figure 1A) and average daily feed intake (ADFI; Figure 1B) compared to the control lambs. LPSH lambs had a higher rectal temperature (Figure 1C) on day 1 (*p* < 0.01) than controls, however, there was no difference among groups in the following days. Lambs administered with LPS had a significant decrease in the number of white blood cells (Figure 2A, *p* < 0.05) in a dose-independent manner on days 1 and 3 after administration, compared with the control group. 

### 3.2. LPS Challenge Induced Systemic Inflammation in Lambs

**Figure 3:**
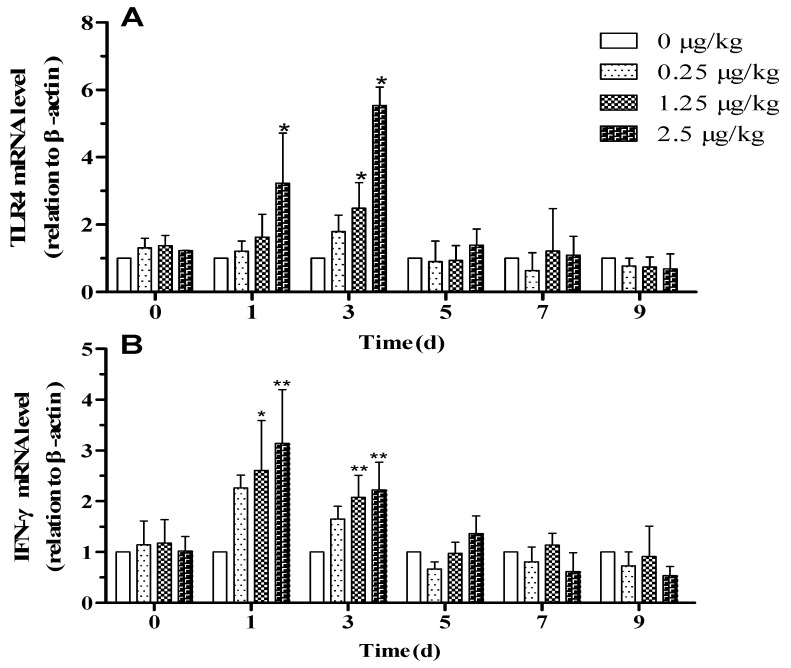
Effect of 0.25, 1.25, and 2.5 μg/kg LPS on expression of inflammatory markers in blood of lambs. (**A**) Toll-like receptor-4 (*TLR4*) and (**B**) Interferon-γ (*IFN-γ*). The values are normalized to control values and expressed as means ± SD. * *p* < 0.05, ** *p* < 0.01, and *** *p* < 0.001, compared with the control group.

Plasma cortisol concentration remained constant on days 0, 1, 3, and 5 in control lambs, but with LPS injection, cortisol concentration increased in a dose-independent manner at 3 h after LPS-challenge on day 1 and returned to pretreatment level on day 5. Following subsequent administration of LPS, plasma cortisol concentration did not differ from the control group (Figure 2B, *p* < 0.05). The expressions of *IFN-γ* and *TLR4* in blood were higher than in the control group after the LPS challenge on days 1 and 3 (Figure 3A–B, *p* < 0.05), in particular in the LPSH group. On days 5, 7, and 9, there was no significant difference between the LPS injected groups and the control group. 

### 3.3. Effect of Resveratrol on Blood Parameters in LPS-Challenged Lambs

**Figure 4:**
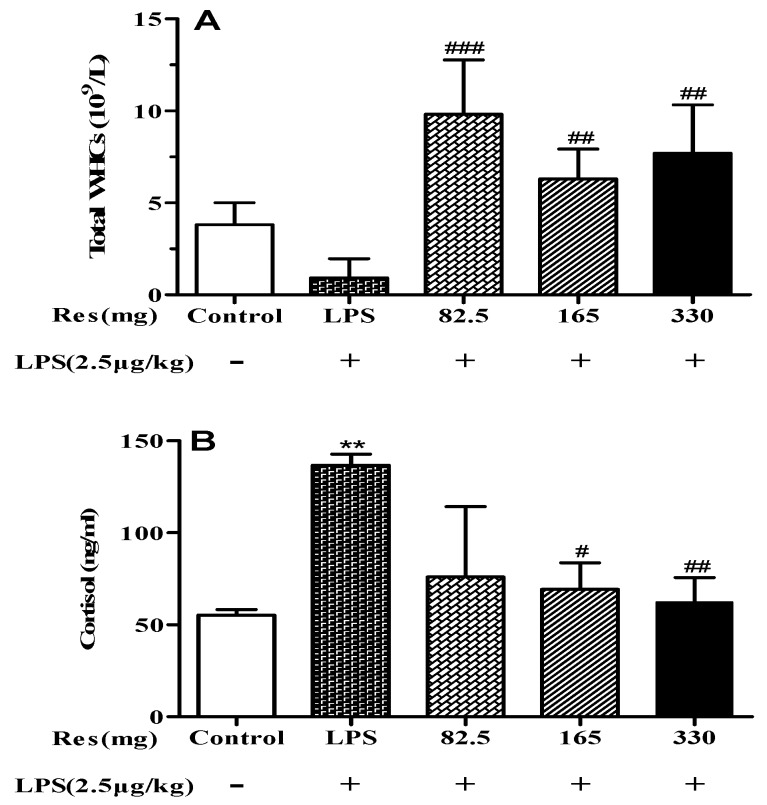
Effect of resveratrol on: (**A**) the number of total white blood cells (WBCs) and (**B**) concentration of plasma cortisol in LPS-challenged lambs. Values are normalized to control values and expressed as means ± SD. * *p* < 0.05, ** *p* < 0.01, and *** *p* < 0.001, compared with the control group; # *p* < 0.05, ## *p* < 0.01, and ### *p* < 0.001, compared with the LPS treatment.

LPS injected lambs had lower WBCs (Figure 4A) and higher concentrations of plasma cortisol (Figure 4B) than the control group. The resveratrol treatment enhanced WBCs and decreased the concentration of plasma cortisol in lambs subjected to an LPS challenge (*p* < 0.05, Figure 4).

### 3.4. Effect of Resveratrol on Expression of Genes Following the LPS Challenge

**Figure 5:**
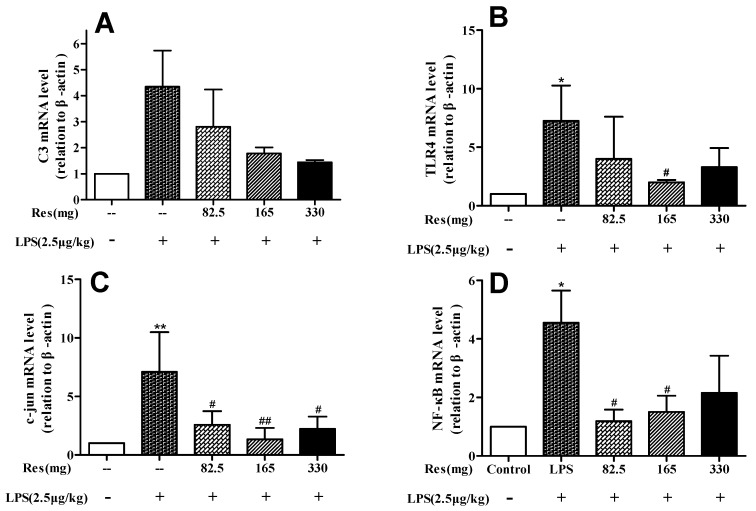
Effect of resveratrol on expression of immune gene *C3* (**A**) and inflammatory markers in peripheral WBCs in LPS-challenged lambs, (**B**) *TLR4*, (**C**) *c-jun*, and (**D**) *NF-κB*. Values are normalized to control values and expressed as means ± SD. * *p* < 0.05, ** *p* < 0.01, and *** *p* < 0.001, compared with the control group; # *p* < 0.05, ## *p* < 0.01, and ### *p* < 0.001, compared with the LPS treatment.

When compared to the control group, LPS injected lambs had a tendency to increase the expression levels of *C3*, while lambs with resveratrol treatment had a tendency to reduce the levels (*p* < 0.10; Figure 5A). The mRNA levels of *TLR4* (Figure 5B), *c-jun* (Figure 5C), and *NF-κB* (Figure 5D) in the LPS injected lambs were significantly higher than in the control group (*p* < 0.05 or *p* < 0.01); however, resveratrol at 165 mg suppressed the expression of these genes compared with the LPS group (*p* < 0.05 or *p* < 0.01). 

### 3.5. Effect of Resveratrol on Expression of Inflammatory Cytokines Following LPS Challenge

**Figure 6:**
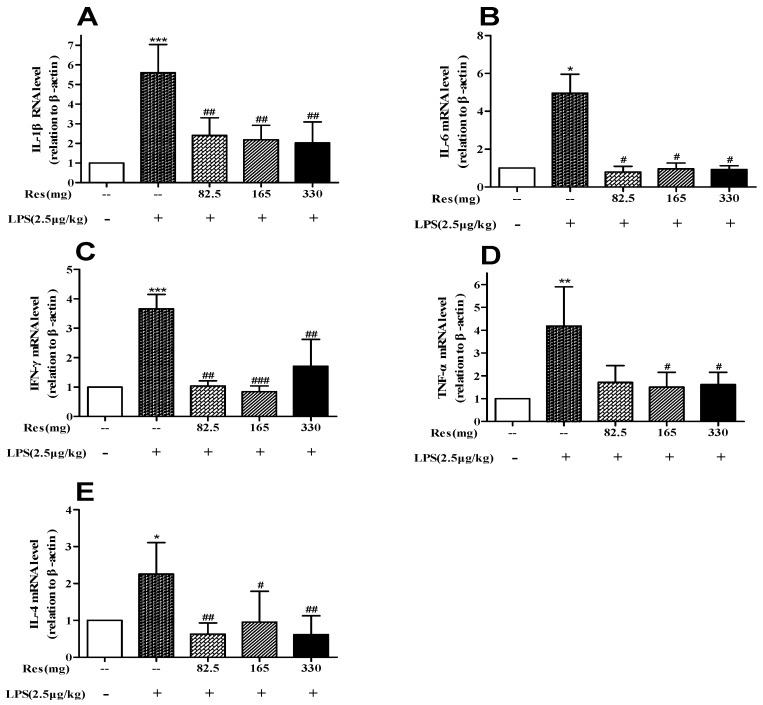
Effect of resveratrol on expression of pro-inflammatory cytokines (**A**) *IL-1β*, (**B**) *IL-6*, (**C**) *IFN-γ*, (**D**) *TNF-α*, and (**E**) anti-inflammatory cytokines *IL-4* in LPS-challenged lambs. The values are normalized to control values and expressed as means ± SD. * *p* < 0.05, ** *p* < 0.01, and *** *p* < 0.001, compared with the control group; # *p* < 0.05, ## *p* < 0.01, and ### *p* < 0.001, compared with the LPS treatment.

LPS administration upregulated the levels of *IL-1β* (*p* < 0.001; Figure 6A), *IL-6* (*p* < 0.05; Figure 6B), *IFN-γ* (*p* < 0.001; Figure 6C), *TNF-α* (Figure 6D; *p* < 0.01), and *IL-4* (*p* < 0.05; Figure 6E). After the administration of 165 mg and 330 resveratrol, the expressions of these genes were lowered significantly.

## 4. Discussion

In recent years, overuse of antibiotics has led to a rise in bacterial resistance to antibiotics, and has become a worldwide concern. Faced with this situation, natural plants are being examined as possible alternatives to antibiotics. Resveratrol has the potential to become an effective natural anti-bacterial drug as it possesses properties of anti-oxidation [13], anti-inflammation [21], anti-platelet aggregation [22,23], microcirculation improvement [24], vascular endothelium protection [25], nervous system protection [26], immunity enhancement, and aging delay. The main finding of this study was that resveratrol provided vital protection against inflammation induced by LPS in lambs. 

### 4.1. LPS Challenge Induced Systemic Inflammation in Lambs

The pathogenesis of many diseases, including inflammatory bowel disease, Crohn’s disease, gout, and cancer, involves inflammation [19]. To reveal the mechanism of inflammatory disease and to find effective counter-measures for the chronic diseases, LPS, as a non-pathogenic immune activator, is being used to establish animal models. An intrajugular injection of 2.5 μg/kg LPS resulted in different expressions of inflammation-related genes in ewes [2]. Yates et al. [27] reported that an intrajugular injection of 0.75 and 1.5 μg/kg LPS caused a dose-dependent inflammatory response, manifested as an increase in rectal temperature, serum cortisol, and serum insulin. A single injection of LPS in animals only induced a short-term acute immune stress, which caused a sharp increase in plasma concentrations of inflammatory cytokines that returned to the pre-injection levels after 4 to 8 h [2,27,28]. Therefore, to reduce the tolerance and avoid the risk of mortality due to LPS in lambs in the present study, an initial dose of 0.25, 1.25, or 2.5 μg/kg LPS was injected every other day with a dose increase of 20% each time to examine the putative effect of resveratrol on inflammatory responses.

LPS induced an inflammatory state in lambs, especially at the high dose of 2.5 µg/kg LPS. The hypothalamic–pituitary–adrenal (HPA) axis is the major system for stress regulation, and cortisol, a hormone from the adrenal cortex, is secreted for its anti-inflammatory actions. In this study, plasma cortisol concentration increased on the first day after administration of LPS then returned to the pre-injection level on the fifth day, which indicated an increased secretion caused by a response of the hypothalamic–pituitary–adrenal axis [29]. The WBCs in the LPSM and LPSH groups were lower than in the control group after administration on days 1, 3, and 5, as was also reported for pigs [4]. According to Wang et al. [17], the reason for blood leukopenia was that the WBCs adhered to the endothelial cells after LPS injection, and the WBCs infiltrated into the tissue through the vascular endothelial barrier. The lower LPS dose (LPSL) did not affect the expressions of *IFN-γ* and *TLR4*, whereas the higher doses (LPSM and LPSH) increased them to an even greater extent than the control group after the LPS challenge on days 1 and 3. However, the magnitude of cortisol response and the expressions of *IFN-γ* and *TLR4* to LPS administration were attenuated with successive injections. This might be due to a lower amplitude of response, relative to the first challenge, and could be associated with the instability and short half-life of inflammatory factors in blood [4]. These findings demonstrated an adaptive endocrine response and an activation of intracellular signaling pathways to repeated inflammatory stimuli of LPS. 

### 4.2. Resveratrol Improves Systemic Inflammation Response in LPS-Injected Lambs

The complement system begins with the formation of antigen–antibody immune complexes, thereby creating a chemo-attractant for immune cells, leading to a release of proteolytic enzymes from intracellular granules, and modulation of cytokine production [30]. The complement component C3 is the central factor in all three activation pathways (classical, alternative, and lectin) [31]. Hadfield et al. [2] reported that the mRNA level *C3* in blood of ewes increased for 6 h after LPS administration, but there was no change in *C3* at 0–4 h. In this study, LPS had a tendency to increase the expression level of *C3* when compared to the control group, whereas lambs treated with resveratrol had a tendency to reduce the level of *C3*. It was reported that the enhanced transcription of the *C3* gene induced by LPS is targeted by the *c-jun* transcription factor [32]. *C-jun* is a component of the *AP-1* transcription complex, which is considered to be the major target and key factor in inflammatory diseases that regulate inflammatory processes. The activity of *AP-1* is regulated by mitogen-activated protein kinase (MAPK) [33]. In the present study, the mRNA level of *c-jun* was enhanced substantially after LPS treatment in the lambs; however, this up-regulation was suppressed markedly by resveratrol. The dose of 165 mg resveratrol showed the best results, indicating that the proper dose of resveratrol is important in alleviating inflammation by the MAPK signal pathway. Studies in rodents also demonstrated resveratrol-mediated protection against LPS-induced inflammation [34,35], which suggests that resveratrol is not species specific in its actions and can be used, most likely, on all animals.

Previous research demonstrated that the inflammatory response to LPS was associated with activation of toll-like receptors (TLRs) whose family members are receptors of the innate immune system that recognize pathogen-associated molecular patterns. Among them, *TLR4* is considered to be a major LPS signaling receptor [36]. Stimulation of *TLR4* triggers activation of transcription factors, which, in turn, enhance the synthesis of inflammatory genes, including cytokines. The formation of an LPS-TLR4/MD-2 complex and the subsequent recruitment of MyD88 adaptor protein induces activation of MAPKs, as well as the transcriptional *NF-κB*, leading to the secretion of various inflammatory mediators [36]. *NF-κB* is present in the cytoplasm as a hetero-trimeric complex composed of three subunits: p50, p65, and *IκB*. After activation of the complex, phosphorylation and degradation of *IκB* exposes nuclear localization signals on the p50/p65 complex, leading to their nuclear translocation and binding to the specific regulated sequences in the DNA, thus controlling gene transcription [37]. *NF-κB* signaling is an inducer of LPS, as well as MAPK signaling, while the attenuation of this signaling by resveratrol contributes to its anti-inflammatory effect [10,38]. *NF-κB* and *AP-1* were key downstream factors of *TLR4* and resveratrol was shown to block the expression of *TLR4* and suppress the phosphorylation of NF-κB and expression of *AP-1* in high glucose-induced mesangial cells [39]. In this study, resveratrol prevented *NF-κB* and *TLR4* gene upregulation caused by LPS. 

The production of inflammatory cytokines such as *IL-1β*, *IL-4*, *IL-6*, *TNF-α*, and *IFN-γ* have strong influence on inflammatory responses and serve as markers in LPS-induced inflammation [40,41,42,43]. *TNF-α* acts as a first signal that enhances the production of other pro-inflammatory cytokines such as *IL-1β*. *IL-1β*, a subtype of *IL-1*, is thought to mediate inflammatory actions by inducing the expression of pro-inflammatory genes and the production of secondary cytokines [44]. In contrast, *IL-4* is often associated with suppression or reduction of inflammatory responses, mainly by inhibiting the secretion of *IL-1β*, *IL-6*, and *TNF-α* [45,46]. Studies have shown that resveratrol regulates the expression of cytokines induced by LPS through the NF-κB signaling pathway. It can activate transcription and promote production of *TNF-α*, *IL-1β*, *IL-4*, *IL-6*, and *IFN-γ* genes. Then *NF-κB* is activated again, which leads to further amplification of the initial inflammatory signal, aggravating the body damage and microcirculatory disorders [37]. In the present study, resveratrol at 165 and 330 mg suppressed the secretion of cytokines, and showed a positive trend to narrow the gap caused by LPS, which indicated that resveratrol regulated immune abnormalities caused by LPS.

## 5. Conclusions

Resveratrol attenuated the LPS-evoked inflammatory activation in lambs by suppressing expression levels of pro-inflammatory cytokines such as *IL-1β*, *IL-6*, *IFN-γ*, and *TNF-α* and anti-inflammatory cytokines *IL-4*, as well as the release of cortisol and the reduction of leucocytes. Resveratrol induced anti-inflammation actions by decreasing complement activity and blocking NF-κB and MAPK signaling pathways. Based on these studies, resveratrol has the potential to be a promising therapeutic reagent for multiple inflammatory illnesses caused by immune stress. Perhaps it can be added to feed in intensively-raised livestock systems, but this still warrants further investigation. 

## Figures and Tables

**Table 1:** Ingredient and nutrient levels of the diet offered the lambs in Experiments 1 and 2.

Ingredient		Nutrient ^2^	
Ingredient, % of DM			
Oat hay	25.00	ME (MJ/kg)	9.50
Corn stalk	15.00	Crude protein (%)	10.89
Corn	31.50	Neutral detergent fiber (%)	43.83
Soybean meal	4.00	Acid detergent fiber (%)	16.71
Wheat bran	4.50	Ca (%)	0.63
DDGS	3.00	P (%)	0.45
Molasses	3.00		
Barley grain	8.50		
NaCl	0.50		
CaHPO_4_	0.80		
Limestone	0.70		
Soybean oil	1.50		
Sodium bicarbonate	1.00		
Premix ^1^	1.00		

DM: Dry Matter; ME: Metabolic Energy; DDGS: Distillers Dried Grains with Solubles. ^1^ The premix provided the following per kg of diets: Vit A, 12 000 IU; Vit D, 2 000 IU; Vit E, 30 IU; Cu, 12 mg; Fe, 64 mg; Mn, 56 mg; Zn, 60 mg; I, 1.2 mg; Se, 0.4 mg; Co, 0.4 mg. ^2^ ME was a calculated value, while the other nutrient levels were measured values.

**Table 2:** List of primer sequences for quantitative real-time PCR.

Gene	Forward Primer 5’-3’	Reverse Primer 5’-3’	Product Length	Annealing Temperature (℃)
*C3*	GCACTGTCCACCAACCTCA	ATCAGGCTTCTGCTTCTCCA	87	58
*TLR4*	GGCATCATCTTCATCGTCCT	CCACTCCAGGTAGGTGTTCC	99	58
*NF-κB*	CATCAGCCAGCGCATCCAGAC	GCACGGCATTCAGGTCGTAGTC	86	61
*c-jun*	AGCGGATCAAGGCGGAGAGG	CCTGAGCATATTGGCGGTGGAC	155	55
*IL-1β*	GCAGGCAGTGTCGGTCATCG	CCTCAGGTCATCATCACGGAAGAC	82	58
*IL-4*	GCGGACTTGACAGGAATCTCAGC	CAGCGTACTTGTACTCGTCTTGGC	80	63
*IL-6*	ACACTGACATGCTGGAGAAGATGC	GCCGCAGCTACTTCATCCGAATAG	132	61
*IFN-R*	ATGTTTCATTTGCCACCATCC	GGTTACGCTTGCTTTGCCTTATGT	81	60
*TNF-α*	CTGGCGGAGGAGGTGCTCTC	GGAGGAAGGAGAAGAGGCTGAGG	85	59
*β-actin*	AGCCTTCCTTCCTGGGCATGGA	GGACAGCACCGTGTTGGCGTAGA	113	60

## Supplementary Materials

The following are available online at https://www.mdpi.com/2076-2615/9/11/872/s1, Table S1: Effect of lipopolysaccharides (LPS) on Heart rate in lambs
