# Supplementary material for: Protective Effect of Resveratrol Improves Systemic Inflammation Responses in LPS-Injected Lambs
Source: Animals (Basel). 2019 Oct 28;9(11):872. doi: 10.3390/ani9110872 (PMC6912468; doi:10.3390/ani9110872)
Supplement: Supplementary file 1 [file animals-09-00872-s001.docx]

**Table S1** Effect of lipopolysaccharides (LPS) on Heart rate in lambs

| **Time (d)** | **LPS (μg/kg BW)** | | | | ***p*-Value** |
| --- | --- | --- | --- | --- | --- |
|  | **0** | **0.25** | **1.25** | **2.5** |  |
| 1 | 104 ± 5.72^a^ | 118 ± 4.08^ab^ | 125 ± 5.12^ab^ | 131 ± 13.73^b^ | ***p*** < 0.05 |
| 3 | 115 ± 4.37^a^ | 120 ± 9.70^ab^ | 137 ± 5.17^b^ | 139 ± 3.17^b^ | ***p*** < 0.05 |
| 5 | 117 ± 4.51^a^ | 120 ± 6.34^a^ | 125 ± 10.09^ab^ | 143 ± 1.20^b^ | ***p*** < 0.05 |
| 7 | 113 ± 6.35^a^ | 134 ± 7.71^ab^ | 133 ± 11.16^ab^ | 144 ± 2.08^b^ | ***p*** < 0.05 |
| 9 | 109 ± 5.01^a^ | 125 ± 8.05^ab^ | 131 ± 16.09^ab^ | 141 ± 2.50^b^ | ***p*** < 0.05 |

^a,b^ Values within a row with different superscripts are different from each other (***p*** < 0.05).
